# Supplementary material for: Wild Bird Surveillance in the Gauteng Province of South Africa during the High-Risk Period for Highly Pathogenic Avian Influenza Virus Introduction
Source: Viruses. 2022 Sep 13;14(9):2027. doi: 10.3390/v14092027 (PMC9504564; doi:10.3390/v14092027)
Supplement: Supplementary file 1 [file viruses-14-02027-s001.zip › Supplemental Figure.pdf]

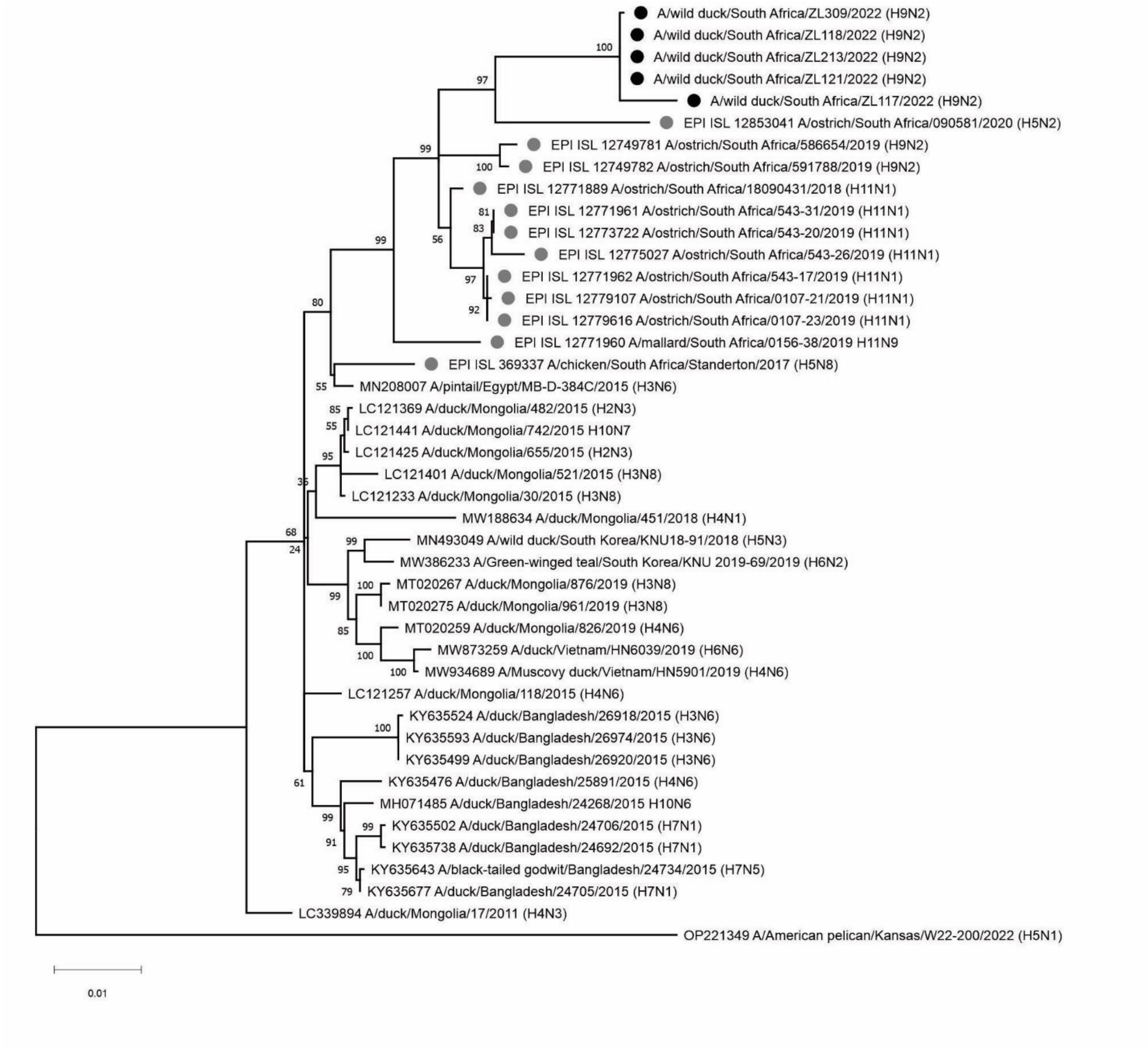

Figure S1 (a). Maximum likelihood phylogenetic tree of the PB2 genes (2,153 bp alignment). Viral sequences from the present study are indicated with black dots, with prior South African virus sequences indicated by grey dots.

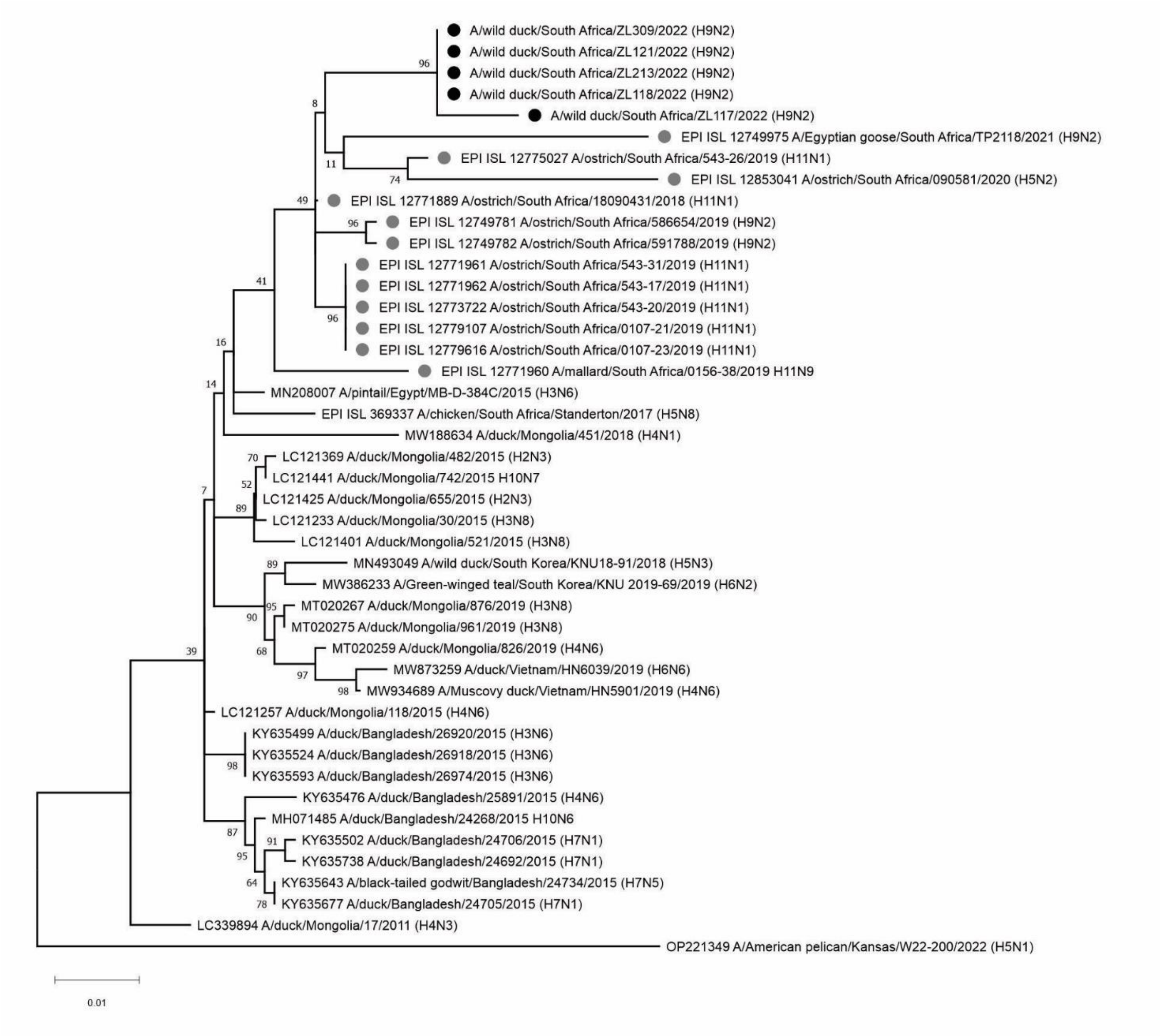

Figure S1 (b). Maximum likelihood phylogenetic tree of the PB2 genes (839 bp alignment). Viral sequences from the present study are indicated with black dots, with prior South African virus sequences indicated by grey dots.

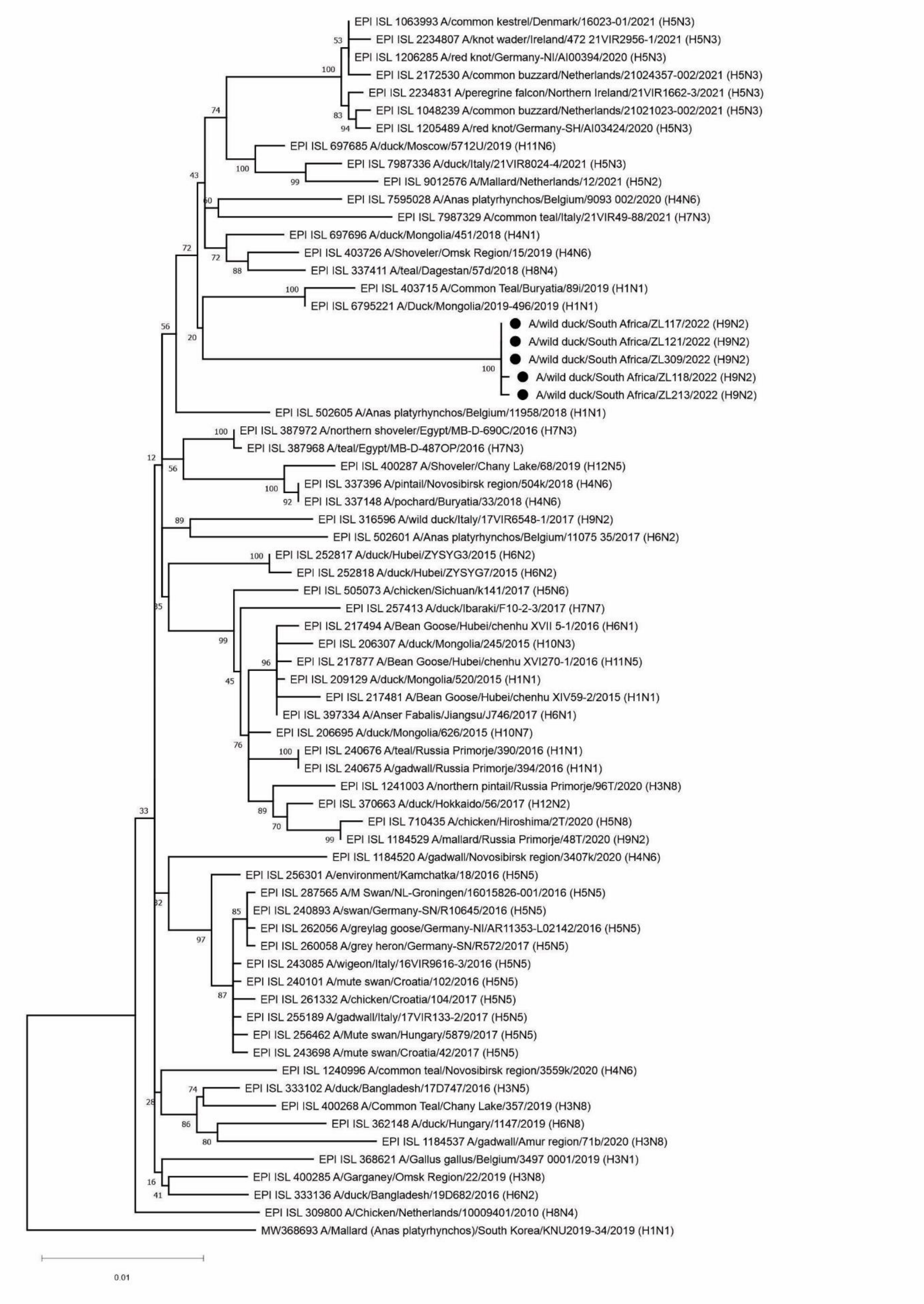

Figure S2. Maximum likelihood phylogenetic tree of the PB1 genes (2,271 bp alignment). Viral sequences from the present study are indicated with black dots.

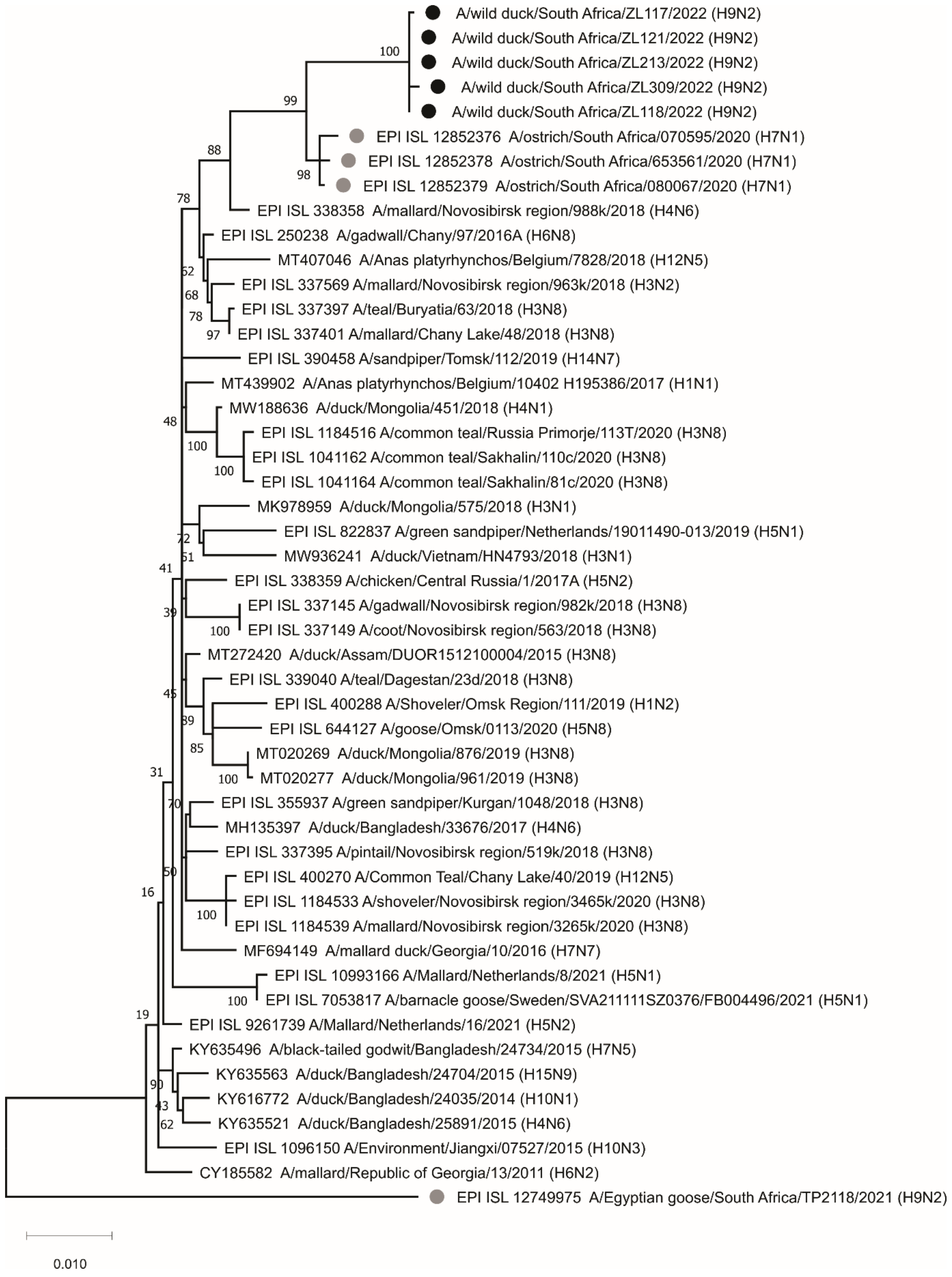

Figure S3. Maximum likelihood phylogenetic tree of the PA genes (1,940 bp alignment). Viral sequences from the present study are indicated with black dots, with prior South African virus sequences indicated by grey dots.

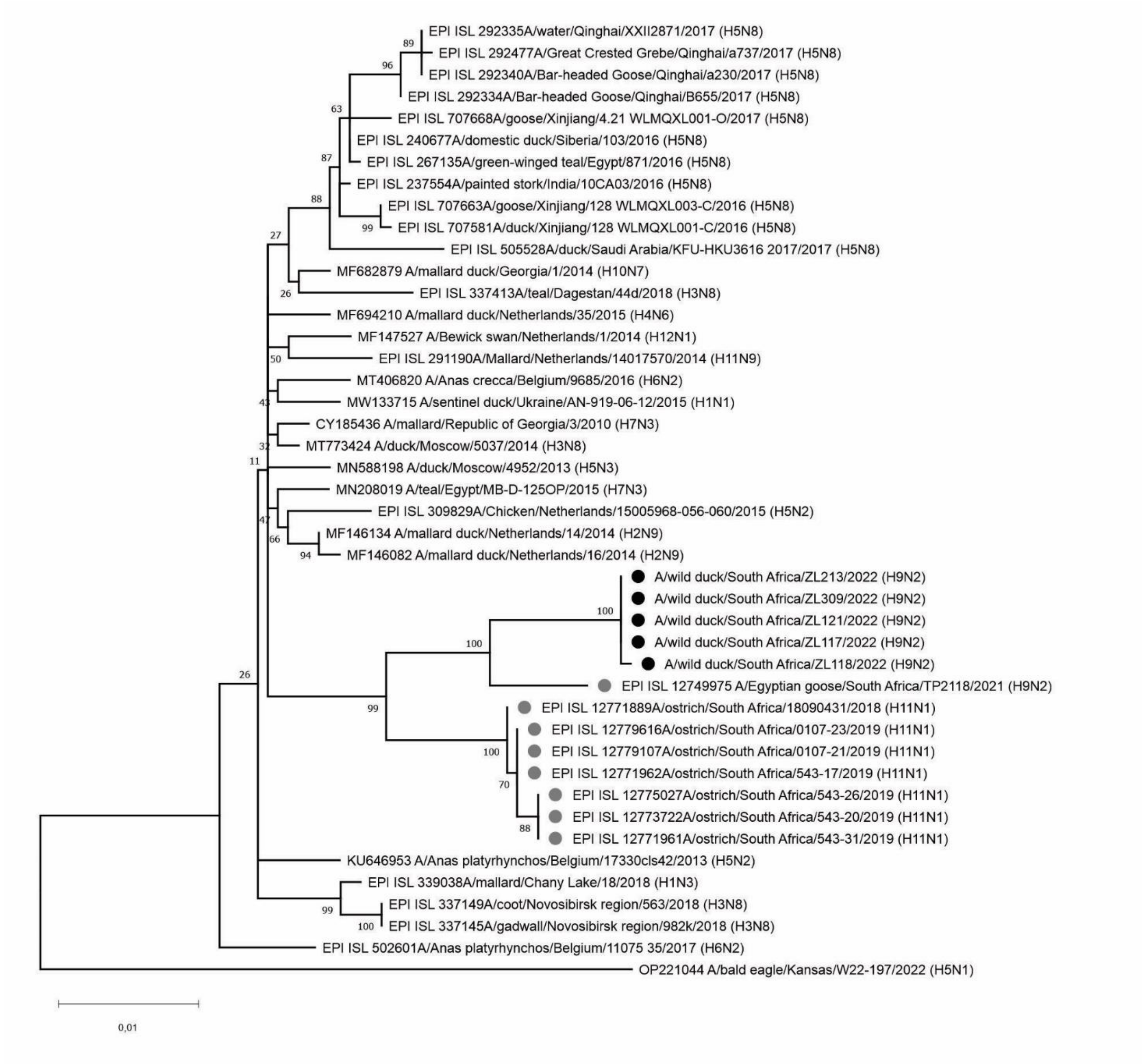

Figure S4. Maximum likelihood phylogenetic tree of the NP genes (1,372 bp alignment). Viral sequences from the present study are indicated with black dots, with prior South African virus sequences indicated by grey dots.

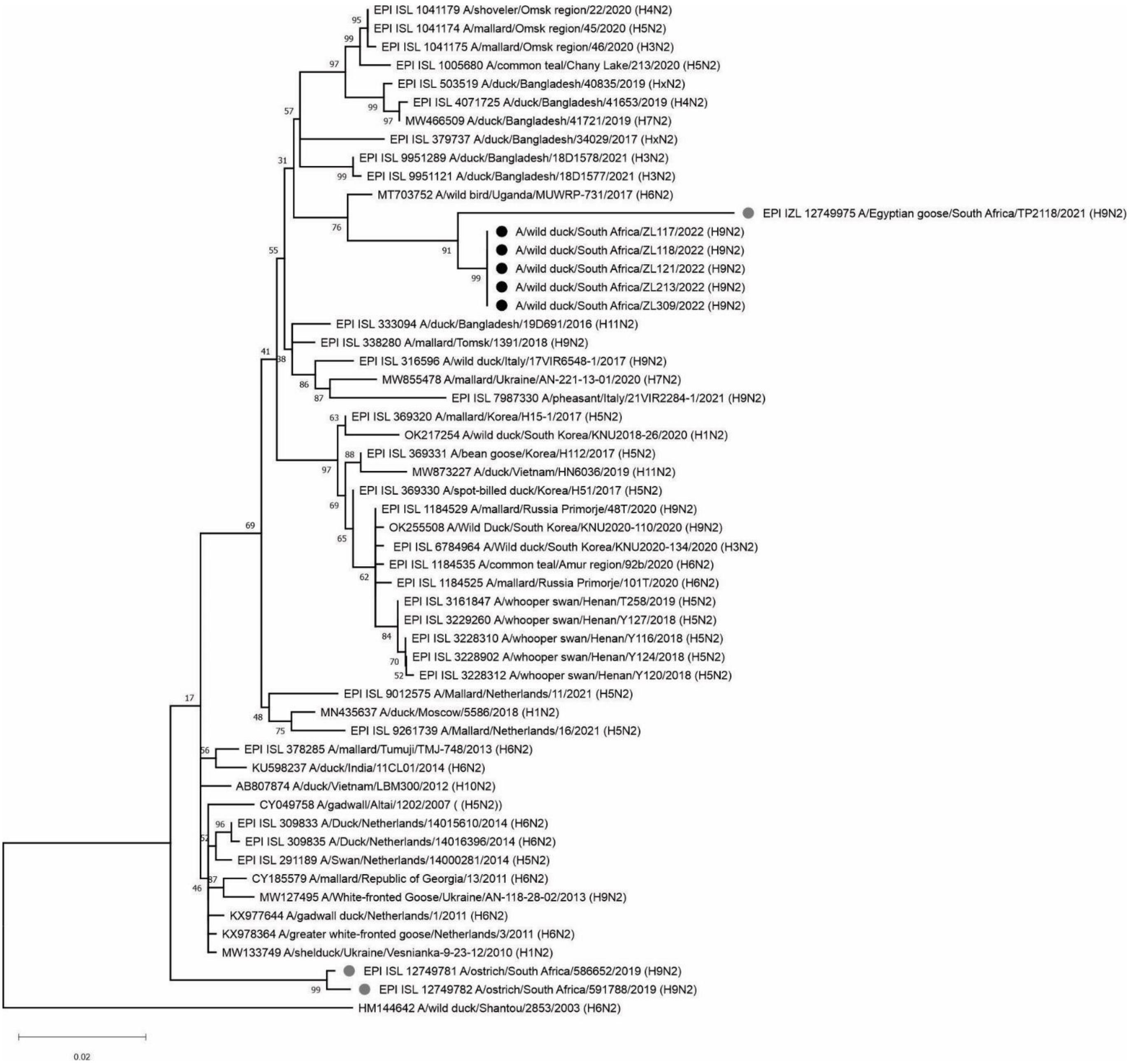

Figure S5. Maximum likelihood phylogenetic tree of the N2 subtype NA genes (902 bp alignment). Viral sequences from the present study are indicated with black dots, with prior South African virus sequences indicated by grey dots.

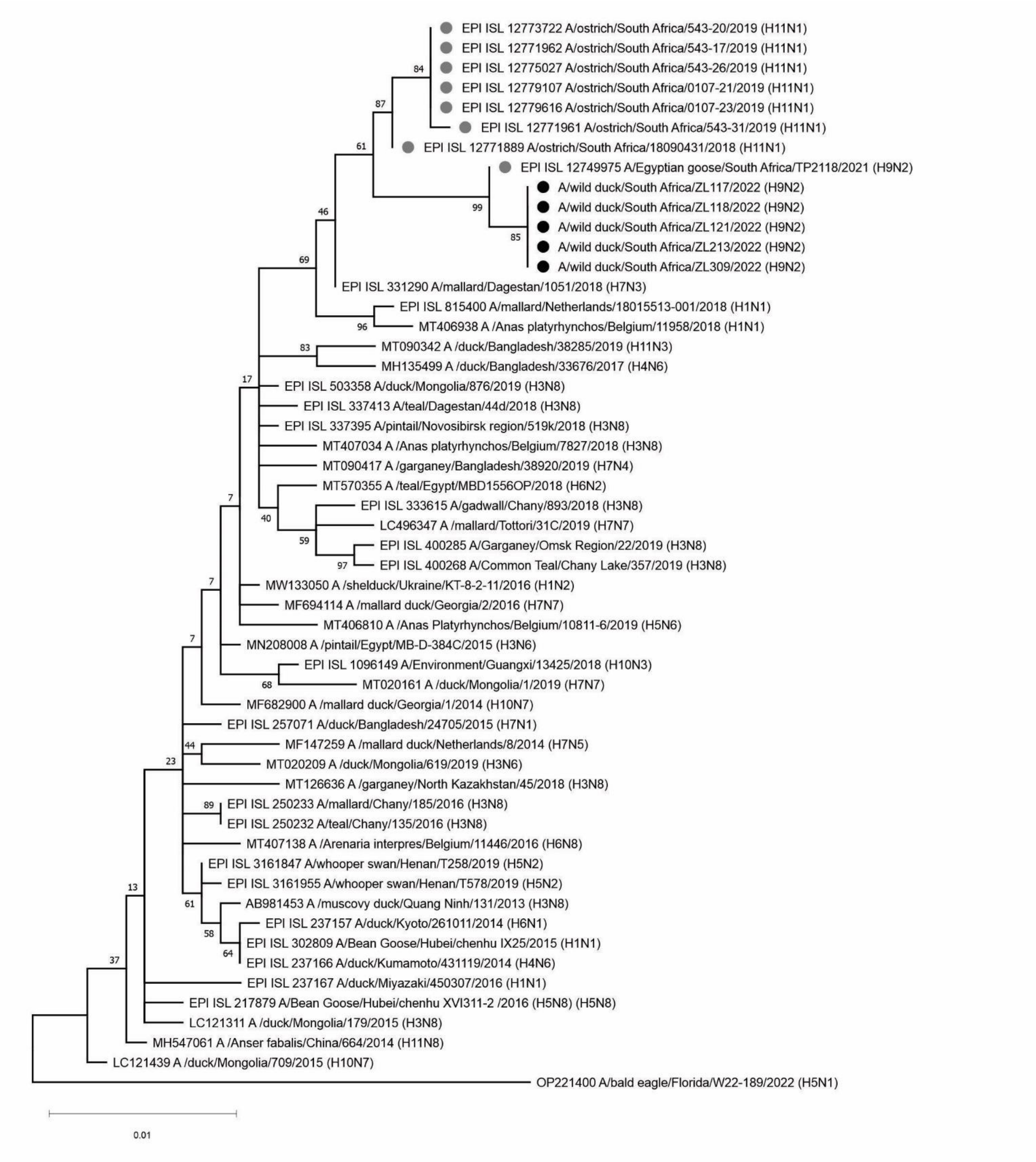

Figure S6. Maximum likelihood phylogenetic tree of the M genes (979 bp alignment). Viral sequences from the present study are indicated with black dots, with prior South African virus sequences indicated by grey dots.

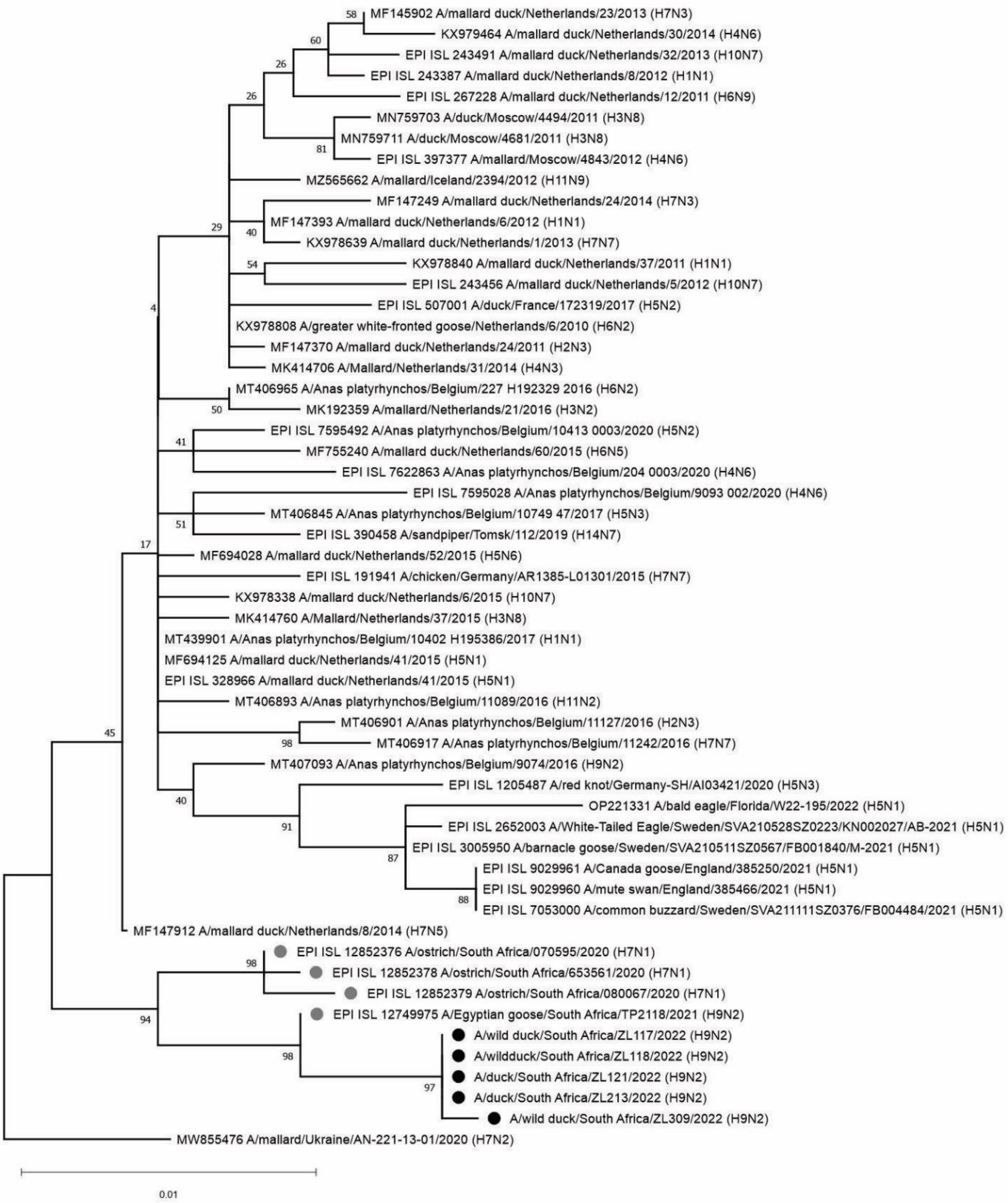

Figure S7. Maximum likelihood phylogenetic tree of the NS genes (832 bp alignment). Viral sequences from the present study are indicated with black dots, with prior South African virus sequences indicated by grey dots.
