# Supplementary material for: Wild Bird Surveillance in the Gauteng Province of South Africa during the High-Risk Period for Highly Pathogenic Avian Influenza Virus Introduction
Source: Viruses. 2022 Sep 13;14(9):2027. doi: 10.3390/v14092027 (PMC9504564; doi:10.3390/v14092027)
Supplement: Supplementary file 1 [file viruses-14-02027-s001.zip › Supplemental Table S1.pdf]

Supplemental Table S1. Samples analysed in the study and summary of results

Sample #	Sampling date	Location	Predominant species observed	Real-time RT-PCR results (Ct value)			Subtype[a]
				IAV group	H5 subtype	H9 subtype	
D1725-2	03-Feb-22	Rooikraal, Heidelberg	SI, EG, WFD	not detected			
D1725-1	03-Feb-22	Rooikraal, Heidelberg	SI, EG, WFD	Positive (37.06)	not detected	not detected	
D1721-1	04-Feb-22	Grootvaly, Springs	SI, EG, WFD	Positive (38.47)	not detected	not detected	
D1726-2	04-Feb-22	Grootvaly, Springs	SI, EG, WFD	not detected			
BA101	08-Feb-22	Bon Accord Dam, Pretoria	EG, SI, EWS	not detected			
BA102	08-Feb-22	Bon Accord Dam, Pretoria	EG, SI, EWS	not detected			
BA103	08-Feb-22	Bon Accord Dam, Pretoria	EG, SI, EWS	Positive (39.57)	not detected	not detected	
BA104	08-Feb-22	Bon Accord Dam, Pretoria	EG, SI, EWS	Positive (39.24)	not detected	not detected	
BA105	08-Feb-22	Bon Accord Dam, Pretoria	EG, SI, EWS	not detected			
BA106	08-Feb-22	Bon Accord Dam, Pretoria	EG, SI, EWS	not detected			
BA107	08-Feb-22	Bon Accord Dam, Pretoria	EG, SI, EWS*	Positive (18.53)	Positive (17.74)	not detected	**H5N1 HPAI[b,c]**
BA108	08-Feb-22	Bon Accord Dam, Pretoria	EG, SI, EWS	not detected			
BA109	08-Feb-22	Bon Accord Dam, Pretoria	EG, SI, EWS	Positive (37.16)	not detected	not detected	
BA110	08-Feb-22	Bon Accord Dam, Pretoria	EG, SI, EWS	not detected			
BA111	08-Feb-22	Bon Accord Dam, Pretoria	EG, SI, EWS	Positive (37.43)	not detected	not detected	
BA112	08-Feb-22	Bon Accord Dam, Pretoria	EG, SI, EWS	not detected			
BA113	08-Feb-22	Bon Accord Dam, Pretoria	EG, SI, EWS	not detected			
BA114	08-Feb-22	Bon Accord Dam, Pretoria	EG, SI, EWS	Positive (36.79)	not detected	not detected	
BA115	08-Feb-22	Bon Accord Dam, Pretoria	EG, SI, EWS	not detected			
BA116	08-Feb-22	Bon Accord Dam, Pretoria	EG, SI, EWS	not detected			
BA117	08-Feb-22	Bon Accord Dam, Pretoria	EG, SI, EWS	not detected			
LC01	09-Feb-22	Loch Vaal, Vereeniging	EG	not detected			
LC02	09-Feb-22	Loch Vaal, Vereeniging	EG	Positive (37.02)	not detected	not detected	
IRN22-001	10-Feb-22	Irene, Centurion	EG, YBD, SI, HI	Positive (35.87)	not detected	not detected	**H8Nx**
IRN22-002	10-Feb-22	Irene, Centurion	EG, YBD, SI, HI	Positive (36.25)	not detected	not detected	
IRN22-003	10-Feb-22	Irene, Centurion	EG, YBD, SI, HI	Positive (37.28)	not detected	not detected	
IRN22-004	10-Feb-22	Irene, Centurion	EG, YBD, SI, HI	not detected			

IRN22-005	10-Feb-22	Irene, Centurion	EG, YBD, SI, HI	Positive (34.44)	not detected	not detected	**H8Nx**
IRN22-006	10-Feb-22	Irene, Centurion	EG, YBD, SI, HI	Positive (36.74)	not detected	not detected	
IRN22-007	10-Feb-22	Irene, Centurion	EG, YBD, SI, HI	Positive (37.66)	not detected	not detected	
IRN22-008	10-Feb-22	Irene, Centurion	EG, YBD, SI, HI	not detected			
IRN22-009	10-Feb-22	Irene, Centurion	EG, YBD, SI, HI	Positive (37.36)	not detected	not detected	
IRN22-010	10-Feb-22	Irene, Centurion	EG, YBD, SI, HI	Positive (36.15)	not detected	not detected	**H8Nx**
IRN22-011	10-Feb-22	Irene, Centurion	EG, YBD, SI, HI	Positive (36.6)	not detected	not detected	
IRN22-012	10-Feb-22	Irene, Centurion	EG, YBD, SI, HI	Positive (37.08)	not detected	not detected	
IRN22-013	10-Feb-22	Irene, Centurion	EG, YBD, SI, HI	Positive (35.06)	not detected	not detected	**H8Nx**
ZL103	11-Feb-22	Zoo Lake, Johannesburg	EG, SI	not detected			
ZL104	11-Feb-22	Zoo Lake, Johannesburg	EG, SI	not detected			
ZL105	11-Feb-22	Zoo Lake, Johannesburg	EG, SI	not detected			
ZL106	11-Feb-22	Zoo Lake, Johannesburg	EG, SI	not detected			
ZL107	11-Feb-22	Zoo Lake, Johannesburg	EG, SI	Positive (35.35)	not detected	not detected	
ZL108	11-Feb-22	Zoo Lake, Johannesburg	EG, SI	not detected			
ZL109	11-Feb-22	Zoo Lake, Johannesburg	EG, SI	Positive (36.22)	not detected	not detected	
ZL110	11-Feb-22	Zoo Lake, Johannesburg	EG, SI	Positive (37.21)	not detected	not detected	
ZL111	11-Feb-22	Zoo Lake, Johannesburg	EG, SI	not detected			
ZL112	11-Feb-22	Zoo Lake, Johannesburg	EG, SI	Positive (35.82)	not detected	not detected	
ZL113	11-Feb-22	Zoo Lake, Johannesburg	EG, SI	not detected			
ZL114	11-Feb-22	Zoo Lake, Johannesburg	EG, SI	not detected			
ZL115	11-Feb-22	Zoo Lake, Johannesburg	EG, SI	not detected			
ZL116	11-Feb-22	Zoo Lake, Johannesburg	EG, SI	not detected			
ZL117	11-Feb-22	Zoo Lake, Johannesburg	EG, SI	Positive (26.84)	not detected	Positive (24.71)	**H9N2**[b]
ZL118	11-Feb-22	Zoo Lake, Johannesburg	EG, SI	Positive (27.55)	not detected	Positive (25.17)	**H9N2** [b]
ZL119	11-Feb-22	Zoo Lake, Johannesburg	EG, SI	Positive (35.35)	not detected	Positive (35.85)	**H9N2**
ZL120	11-Feb-22	Zoo Lake, Johannesburg	EG, SI	Positive (36.95)	not detected	not detected	
ZL121	11-Feb-22	Zoo Lake, Johannesburg	EG, SI	Positive (24.30)	not detected	Positive (24.48)	**H9N2**[b]
ZL122	11-Feb-22	Zoo Lake, Johannesburg	EG, SI	not detected			
BA 201	15-Feb-22	Bon Accord Dam, Pretoria	SI, WCE	not detected			
BA 202	15-Feb-22	Bon Accord Dam, Pretoria	SI, WCE	not detected			

BA 203	15-Feb-22	Bon Accord Dam, Pretoria	SI, WCE	not detected			
BA 204	15-Feb-22	Bon Accord Dam, Pretoria	SI, WCE	not detected			
BA 205	15-Feb-22	Bon Accord Dam, Pretoria	SI, WCE	not detected			
BA 206	15-Feb-22	Bon Accord Dam, Pretoria	SI, WCE	not detected			
KD 003	16-Feb-22	Klipdrift Dam, Randfontein	WFD, SI	not detected			
KD 001	16-Feb-22	Klipdrift Dam, Randfontein	WFD, SI	not detected			
KD 002	16-Feb-22	Klipdrift Dam, Randfontein	WFD, SI	Positive (34.83)	not detected	not detected	**H3N1**
ZL101	17-Feb-22	Zoo Lake, Johannesburg	EG, SI	not detected			
ZL201	17-Feb-22	Zoo Lake, Johannesburg	EG, SI	not detected			
ZL202	17-Feb-22	Zoo Lake, Johannesburg	EG, SI	not detected			
ZL203	17-Feb-22	Zoo Lake, Johannesburg	EG, SI	not detected			
ZL204	17-Feb-22	Zoo Lake, Johannesburg	EG, SI	Positive (33.55)	not detected	not detected	
ZL205	17-Feb-22	Zoo Lake, Johannesburg	EG, SI	Positive (37.08)	not detected	not detected	
ZL206	17-Feb-22	Zoo Lake, Johannesburg	EG, SI	Positive (34.96)	Positive (34.02)	not detected	**H5N2**[b]
ZL207	17-Feb-22	Zoo Lake, Johannesburg	EG, SI	not detected			
ZL208	17-Feb-22	Zoo Lake, Johannesburg	EG, SI	not detected			
ZL209	17-Feb-22	Zoo Lake, Johannesburg	EG, SI	not detected			
ZL210	17-Feb-22	Zoo Lake, Johannesburg	EG, SI	Positive (37.80)	not detected	not detected	
ZL211	17-Feb-22	Zoo Lake, Johannesburg	EG, SI	Positive (36.91)	not detected	not detected	
ZL212	17-Feb-22	Zoo Lake, Johannesburg	EG, SI	not detected			
ZL213	17-Feb-22	Zoo Lake, Johannesburg	EG, SI	Positive (33.95)	not detected	Positive (36.44)	**H9N2**[b]
ZL214	17-Feb-22	Zoo Lake, Johannesburg	EG, SI	not detected			
ZL215	17-Feb-22	Zoo Lake, Johannesburg	EG, SI	not detected			
ZK001	18-Feb-22	Zeekoegat, Roodeplaat	EG	Positive (37.73)	not detected	not detected	
ZK002	18-Feb-22	Zeekoegat, Roodeplaat	EG	not detected			
ZK003	18-Feb-22	Zeekoegat, Roodeplaat	EG	not detected			
ZL102	18-Feb-22	Zoo Lake, Johannesburg	EG, SI	not detected			
BA301	22-Feb-22	Bon Accord Dam, Pretoria	EG, SI, EWS, SWG, BLP, BH	not detected			
BA302	22-Feb-22	Bon Accord Dam, Pretoria	EG, SI, EWS, SWG, BLP, BH	not detected			
BA303	22-Feb-22	Bon Accord Dam, Pretoria	EG, SI, EWS, SWG, BLP, BH	Positive (37.07)	Positive (36.95)	not detected	**H5Nx**[c]

BA304	22-Feb-22	Bon Accord Dam, Pretoria	EG, SI, EWS, SWG, BLP, BH	not detected			
BA305	22-Feb-22	Bon Accord Dam, Pretoria	EG, SI, EWS, SWG, BLP, BH	not detected			
BA306	22-Feb-22	Bon Accord Dam, Pretoria	EG, SI, EWS, SWG, BLP, BH	not detected			
BA307	22-Feb-22	Bon Accord Dam, Pretoria	EG, SI, EWS, SWG, BLP, BH	Positive (36.96)	not detected	not detected	
BA308	22-Feb-22	Bon Accord Dam, Pretoria	EG, SI, EWS, SWG, BLP, BH	not detected			
BA309	22-Feb-22	Bon Accord Dam, Pretoria	EG, SI, EWS, SWG, BLP, BH	not detected			
BA310	22-Feb-22	Bon Accord Dam, Pretoria	EG, SI, EWS, SWG, BLP, BH	not detected			
TRF001	23-Feb-22	Turffontein, Johannesburg	EWS, SI	Positive (29.29)	Positive (33.34)	not detected	**H5N1 HPAI**[e]
TRF002	23-Feb-22	Turffontein, Johannesburg	EWS, SI	Positive (35.54)	not detected	not detected	
TRF003	23-Feb-22	Turffontein, Johannesburg	EWS, SI	not detected			
TRF004	23-Feb-22	Turffontein, Johannesburg	EWS, SI	Positive (34.35)	not detected	not detected	
TRF005	23-Feb-22	Turffontein, Johannesburg	EWS, SI	Positive (37.98)	not detected	not detected	
WPN001	23-Feb-22	Wemmerpan, Johannesburg	EG, SI	not detected			
WPN002	23-Feb-22	Wemmerpan, Johannesburg	EG, SI	not detected			
WPN003	23-Feb-22	Wemmerpan, Johannesburg	EG, SI	Positive (35.66)	not detected	not detected	
WPN004	23-Feb-22	Wemmerpan, Johannesburg	EG, SI	not detected			
WPN005	23-Feb-22	Wemmerpan, Johannesburg	EG, SI	not detected			
WPN006	23-Feb-22	Wemmerpan, Johannesburg	EG, SI	not detected			
WPN007	23-Feb-22	Wemmerpan, Johannesburg	EG, SI	Positive (35.48)	not detected	not detected	
WPN008	23-Feb-22	Wemmerpan, Johannesburg	EG, SI	not detected			
WPN009	23-Feb-22	Wemmerpan, Johannesburg	EG, SI	Positive (36.44)	not detected	not detected	
WPN010	23-Feb-22	Wemmerpan, Johannesburg	EG, SI	Positive (37.64)	not detected	not detected	
ZL301	23-Feb-22	Zoo Lake, Johannesburg	EG, SI	not detected			
ZL302	23-Feb-22	Zoo Lake, Johannesburg	EG, SI	not detected			
ZL303	23-Feb-22	Zoo Lake, Johannesburg	EG, SI	Positive (37.13)	not detected	Positive (35.84)	
ZL304	23-Feb-22	Zoo Lake, Johannesburg	EG, SI	not detected			
ZL305	23-Feb-22	Zoo Lake, Johannesburg	EG, SI	not detected			
ZL306	23-Feb-22	Zoo Lake, Johannesburg	EG, SI	not detected			
ZL307	23-Feb-22	Zoo Lake, Johannesburg	EG, SI	Positive (36.96)	not detected	not detected	
ZL308	23-Feb-22	Zoo Lake, Johannesburg	EG, SI	not detected			

ZL309	23-Feb-22	Zoo Lake, Johannesburg	EG, SI	Positive (25.96)	not detected	Positive (26.01)	**H9N2**[b,c]
ZL310	23-Feb-22	Zoo Lake, Johannesburg	EG, SI	Positive (35.28)	not detected	Positive (36.9)	**H9N2**
IRN24-001	24-Feb-22	Irene, Centurion	EG, YBD, SI, HI	not detected			
IRN24-002	24-Feb-22	Irene, Centurion	EG, YBD, SI, HI	not detected			
IRN24-003	24-Feb-22	Irene, Centurion	EG, YBD, SI, HI	not detected			
IRN24-004	24-Feb-22	Irene, Centurion	EG, YBD, SI, HI	not detected			
IRN24-005	24-Feb-22	Irene, Centurion	EG, YBD, SI, HI	not detected			
IRN24-006	24-Feb-22	Irene, Centurion	EG, YBD, SI, HI	not detected			
IRN24-007	24-Feb-22	Irene, Centurion	EG, YBD, SI, HI	not detected			
IRN24-008	24-Feb-22	Irene, Centurion	EG, YBD, SI, HI	not detected			
IRN24-009	24-Feb-22	Irene, Centurion	EG, YBD, SI, HI	not detected			
IRN24-010	24-Feb-22	Irene, Centurion	EG, YBD, SI, HI	not detected			
BA401	01-Mar-22	Bon Accord Dam, Pretoria	EG, SI, EWS, SWG, BLP, BH	not detected			
BA402	01-Mar-22	Bon Accord Dam, Pretoria	EG, SI, EWS, SWG, BLP, BH	not detected			
BA403	01-Mar-22	Bon Accord Dam, Pretoria	EG, SI, EWS, SWG, BLP, BH	Positive (36.91)	not detected	not detected	
BA404	01-Mar-22	Bon Accord Dam, Pretoria	EG, SI, EWS, SWG, BLP, BH	not detected			
BA405	01-Mar-22	Bon Accord Dam, Pretoria	EG, SI, EWS, SWG, BLP, BH	not detected			
BA406	01-Mar-22	Bon Accord Dam, Pretoria	EG, SI, EWS, SWG, BLP, BH	not detected			
BA407	01-Mar-22	Bon Accord Dam, Pretoria	EG, SI, EWS, SWG, BLP, BH	not detected			
BA408	01-Mar-22	Bon Accord Dam, Pretoria	EG, SI, EWS, SWG, BLP, BH	not detected			
BA409	01-Mar-22	Bon Accord Dam, Pretoria	EG, SI, EWS, SWG, BLP, BH	not detected			
BA410	01-Mar-22	Bon Accord Dam, Pretoria	EG, SI, EWS, SWG, BLP, BH	not detected			
D3396-1	01-Mar-22	Heidelberg Dump, Heidelberg	EG, SI	not detected			
D3396-2	01-Mar-22	Heidelberg Dump, Heidelberg	EG, SI	not detected			
D3397-1	01-Mar-22	Rooikraal, Heidelberg	EG, SI	not detected			
D3397-2	01-Mar-22	Rooikraal, Heidelberg	EG, SI	not detected			
D3397-3	01-Mar-22	Rooikraal, Heidelberg	EG, SI	not detected			
D3398-1	02-Mar-22	Klipdrift Dam, Randfontein	WFD, SI EG	not detected			
D3398-2	02-Mar-22	Klipdrift Dam, Randfontein	WFD, SI, EG	not detected			
D3393	02-Mar-22	Klipdrift Dam, Randfontein	WFD, SI	Positive (35.13)	not detected	not detected	

ZL401	04-Mar-22	Zoo Lake, Johannesburg	EG, SI	not detected			
ZL402	04-Mar-22	Zoo Lake, Johannesburg	EG, SI	Positive (38.17)	not detected	not detected	
ZL403	04-Mar-22	Zoo Lake, Johannesburg	EG, SI	Positive (37.0)	not detected	not detected	
ZL404	04-Mar-22	Zoo Lake, Johannesburg	EG, SI	not detected			
ZL405	04-Mar-22	Zoo Lake, Johannesburg	EG, SI	not detected			
ZL406	04-Mar-22	Zoo Lake, Johannesburg	EG, SI	Positive (36.83)	not detected	not detected	
ZL407	04-Mar-22	Zoo Lake, Johannesburg	EG, SI	Positive (33.35)	not detected	not detected	
ZL408	04-Mar-22	Zoo Lake, Johannesburg	EG, SI	Positive (35.26)	not detected	not detected	
ZL409	04-Mar-22	Zoo Lake, Johannesburg	EG, SI	not detected			
ZL410	04-Mar-22	Zoo Lake, Johannesburg	EG, SI	Positive (35.29)	not detected	Positive (36.64)	**H9N2**
ZL411	04-Mar-22	Zoo Lake, Johannesburg	EG, SI	not detected			
BA501	09-Mar-22	Bon Accord Dam, Pretoria	EG, SI, BH EWS	not detected			
BA502	09-Mar-22	Bon Accord Dam, Pretoria	EG, SI, BH EWS	not detected			
BA503	09-Mar-22	Bon Accord Dam, Pretoria	EG, SI, BH	not detected			
BA504	09-Mar-22	Bon Accord Dam, Pretoria	EG, SI, BH	not detected			
BA505	09-Mar-22	Bon Accord Dam, Pretoria	EG, SI, BH	not detected			
BA506	09-Mar-22	Bon Accord Dam, Pretoria	EG, SI, BH	not detected			
BA507	09-Mar-22	Bon Accord Dam, Pretoria	EG, SI, BH	Positive (36.93)	not detected	not detected	**H4N2**
BA508	09-Mar-22	Bon Accord Dam, Pretoria	EG, SI, BH	not detected			
BA509	09-Mar-22	Bon Accord Dam, Pretoria	EG, SI, BH	not detected			
BA510	09-Mar-22	Bon Accord Dam, Pretoria	EG, SI, BH	not detected			
ZL501	11-Mar-22	Zoo Lake, Johannesburg	EG, SI, BH	Positive (36.23)	not detected	not detected	
ZL502	11-Mar-22	Zoo Lake, Johannesburg	EG, SI, BH	not detected			
ZL503	11-Mar-22	Zoo Lake, Johannesburg	EG, SI, BH	Positive (35.14)	not detected	not detected	
ZL504	11-Mar-22	Zoo Lake, Johannesburg	EG, SI, BH	Positive (36.14)	not detected	not detected	
ZL505	11-Mar-22	Zoo Lake, Johannesburg	EG, SI, BH	Positive (37.25)	not detected	not detected	
ZL506	11-Mar-22	Zoo Lake, Johannesburg	EG, SI, BH	Positive (36.6)	Positive (34.91)	not detected	**H5N1**[d]
ZL507	11-Mar-22	Zoo Lake, Johannesburg	EG, SI, BH	Positive (38.01)	not detected	not detected	
ZL508	11-Mar-22	Zoo Lake, Johannesburg	EG, SI, BH	Positive (35.87)	not detected	not detected	
ZL509	11-Mar-22	Zoo Lake, Johannesburg	EG, SI, BH	Positive (36.84)	not detected	not detected	

ZL510	11-Mar-22	Zoo Lake, Johannesburg	EG, SI, BH	not detected			
D4120-1	14-Mar-22	Baja Dam, Bronkhorstspruit	EG, SI	Positive (36.76)	not detected	not detected	
D4120-2	14-Mar-22	Baja Dam, Bronkhorstspruit	EG, SI	Positive (35.9)	not detected	not detected	
BA601	15-Mar-22	Bon Accord Dam, Pretoria	EG, SI, BH, SWG, WCE, EWS	Positive (35.84)	not detected	not detected	
BA602	15-Mar-22	Bon Accord Dam, Pretoria	EG, SI, BH, SWG, WCE, EWS	Positive (36.86)	not detected	not detected	
BA603	15-Mar-22	Bon Accord Dam, Pretoria	EG, SI, BH, SWG, WCE, EWS	Positive (35.39)	not detected	not detected	
BA604	15-Mar-22	Bon Accord Dam, Pretoria	EG, SI, BH, SWG, WCE, EWS	Positive (35.81)	not detected	not detected	
BA605	15-Mar-22	Bon Accord Dam, Pretoria	EG, SI, BH, SWG, WCE, EWS	Positive (36.23)	not detected	not detected	
BA606	15-Mar-22	Bon Accord Dam, Pretoria	EG, SI, BH, SWG, WCE, EWS	Positive (35.35)	not detected	not detected	
BA607	15-Mar-22	Bon Accord Dam, Pretoria	EG, SI, BH, SWG, WCE, EWS	Positive (35.74)	not detected	not detected	
BA608	15-Mar-22	Bon Accord Dam, Pretoria	EG, SI, BH, SWG, WCE, EWS	not detected			
BA609	15-Mar-22	Bon Accord Dam, Pretoria	EG, SI, BH, SWG, WCE, EWS	Positive (36.58)	not detected	not detected	
BA610	15-Mar-22	Bon Accord Dam, Pretoria	EG, SI, BH, SWG, WCE, EWS	Positive (36.33)	not detected	not detected	
BA611	15-Mar-22	Bon Accord Dam, Pretoria	EG, SI, BH, SWG, WCE, EWS	Positive (36.56)	Positive (35.86)	not detected	**H5N1**[d]
BA612	15-Mar-22	Bon Accord Dam, Pretoria	EG, SI, BH, SWG, WCE, EWS	Positive (37.27)	not detected	not detected	
BA613	15-Mar-22	Bon Accord Dam, Pretoria	EG, SI, BH, SWG, WCE, EWS	Positive (34.86)	not detected	not detected	
D4121-1	15-Mar-22	Loch Vaal, Vereeniging	EG	Positive (36.6)	not detected	not detected	
D4121-2	15-Mar-22	Loch Vaal, Vereeniging	EG	Positive (37.39)	not detected	not detected	
ZL601	18-Mar-22	Zoo Lake, Johannesburg	EG, SI	not detected			
ZL602	18-Mar-22	Zoo Lake, Johannesburg	EG, SI	not detected			
ZL603	18-Mar-22	Zoo Lake, Johannesburg	EG, SI	Positive (37.83)	not detected	not detected	
ZL604	18-Mar-22	Zoo Lake, Johannesburg	EG, SI	not detected			
ZL605	18-Mar-22	Zoo Lake, Johannesburg	EG, SI	Positive (36.61)	not detected	not detected	
ZL606	18-Mar-22	Zoo Lake, Johannesburg	EG, SI	not detected			
ZL607	18-Mar-22	Zoo Lake, Johannesburg	EG, SI	not detected			
ZL608	18-Mar-22	Zoo Lake, Johannesburg	EG, SI	not detected			
ZL609	18-Mar-22	Zoo Lake, Johannesburg	EG, SI	Positive (37.15)	not detected	not detected	
D4383-1	22-Mar-22	Wemmerpan, Johannesburg	EG	Positive (37.06)	not detected	not detected	
D4383-2	22-Mar-22	Wemmerpan, Johannesburg	SI	Positive (35.86)	not detected	not detected	
D5513-1	19-Apr-22	Loch Vaal, Vereeniging	EG	Positive (35.08)	not detected	Positive (35.93)	**H9N2**

D5513-2	19-Apr-22	Loch Vaal, Vereeniging	EG	Positive (34.93)	Positive (36.96)	not detected	**H5Nx[d]**
D5514-1	20-Apr-22	Klipdrift Dam, Randfontein	WFD, SI EG	Not detected			
D5514-2	20-Apr-22	Klipdrift Dam, Randfontein	WFD, SI, EG	Not detected			
D5648-1	21-Apr-22	Leeukop Prison Dam, Randfontein	WFD, SI	Positive (36.86)	not detected	not detected	
D5648-2	21-Apr-22	Leeukop Prison Dam, Randfontein	WFD, SI	Positive (34.64)	not detected	not detected	
D6166-1	03-May-22	Klipdrift Dam, Randfontein	WFD, SI, EG	not detected			
D6166-2	03-May-22	Klipdrift Dam, Randfontein	WFD, SI, EG	Positive (36.05)	not detected	not detected	
D6167-1	04-May-22	Log Vaal, Vereeniging	EG	not detected			
D6167-2	04-May-22	Log Vaal, Vereeniging	EG	not detected			
D6540	09-May-22	Heidelberg Dump, Heidelberg	EG, SI	not detected			
D6539-1	09-May-22	Rooikraal, Heidelberg	EG, SI	not detected			
D6539-2	09-May-22	Rooikraal, Heidelberg	EG, SI	Positive (36.71)	not detected	Positive (37.0)	**H9N2**
D6541-1	11-May-22	Leeukop Prison Dam, Randfontein	WFD, SI	not detected			
D6541-2	11-May-22	Leeukop Prison Dam, Randfontein	WFD, SI	not detected			
D6541-3	11-May-22	Leeukop Prison Dam, Randfontein	WFD, SI	not detected			
D7021-1	12-May-22	Turffontein, Johannesburg	EG, SI	not detected			
D7021-2	12-May-22	Turffontein, Johannesburg	EG, SI	not detected			
D7019-1	16-May-22	Baja Dam, Bronkhorstspruit	EG, SI	Positive (35.53)	not detected	not detected	
D7019-2	16-May-22	Baja Dam, Bronkhorstspruit	EG, SI	not detected			
D7020-1	17-May-22	Grootvaly, Springs	SI, EG, WFD	Positive (37.16)	not detected	not detected	
D7020-2	17-May-22	Grootvaly, Springs	SI, EG, WFD	Positive (35.77)	not detected	not detected	
D7021-1	12-May-22	Turffontein, Johannesburg	EG, SI ,EWS	not detected			
D7021-2	12-May-22	Turffontein, Johannesburg	EG, SI EWS	not detected			
D7427-1	30-May-22	Zoo Lake, Johannesburg	EG, SI	Positive (35.68)	not detected	not detected	
D7427-2	30-May-22	Zoo Lake, Johannesburg	EG, SI	Positive (36.18)	not detected	not detected	

[a]Non- H5/H9 H and -N subtypes determined by subtype-specific SYBR green r RT-PCRs; [b]Complete genome sequence; [c]Virus isolated in cell culture or eggs; [d]Pathotype could not be determined; [e]Sanger DNA sequencing of HA_0=PLREKRRKRGLF; EG- Egyptian Goose, SI-Sacred Ibis, HI- Hadeda Ibis, YBD- Yellow-Billed Duck, WFD- White Faced Duck, EWS- European White Stork, WCE- Western Cattle Egret, SWG- Spur-Winged Goose, BL- Blacksmith Lapwing Plover, BH- Black Heron, sampled from a moribund bird* or carcass**
