# Supplementary material for: Wild Bird Surveillance in the Gauteng Province of South Africa during the High-Risk Period for Highly Pathogenic Avian Influenza Virus Introduction
Source: Viruses. 2022 Sep 13;14(9):2027. doi: 10.3390/v14092027 (PMC9504564; doi:10.3390/v14092027)
Supplement: Supplementary file 1 [file viruses-14-02027-s001.zip › Supplemental Table S2.pdf]

Supplemental Table S2. tMRCA analysis

Gene	Node (X)	Node age	95 % HPD	Posterior probability
**PB2**	A/wild duck/ZL117/2022 (H9N2)<br>X<br>A/wild ducks/ZA/ ZL118/2022 (H9N2)<br>A/wild ducks/ZA/ ZL121/2022 (H9N2)<br>A/wild ducks/ZA/ ZL213/2022 (H9N2)<br>A/wild ducks/ZA/ ZL309/2022 (H9N2)	Jul 2021	Dec 2020-Dec 2021	1
**PB2**	A/wild duck/ZL117/2022 (H9N2)<br>A/wild ducks/ZA/ ZL118/2022 (H9N2)<br>A/wild ducks/ZA/ ZL121/2022 (H9N2)<br>A/wild ducks/ZA/ ZL213/2022 (H9N2)<br>A/wild ducks/ZA/ ZL309/2022 (H9N2)<br>X<br>A/ostrich/ZA/090581/2020 (H5N2)	Jul 2019	Aug 2018-Mar 2020	1
**PB2**	A/ostrich/ZA/586654/2019 (H9N2)<br>A/ostrich/ZA/591788/2019 (H9N2)<br>X<br>A/wild duck/ZL117/2022 (H9N2)<br>A/wild ducks/ZA/ ZL118/2022 (H9N2)<br>A/wild ducks/ZA/ ZL121/2022 (H9N2)<br>A/wild ducks/ZA/ ZL213/2022 (H9N2)<br>A/wild ducks/ZA/ ZL309/2022 (H9N2)<br>A/ostrich/ZA/090581/2020 (H5N2)	Feb 2018	May 2017-Nov 2018	0.39
**PB2**	A/ostrich/ZA/18090431/2018 (H11N1)<br>X<br>A/ostrich/ZA/543-31/2019 (H11N1)<br>A/ostrich/ZA/543-20/2019 (H11N1)<br>A/ostrich/ZA/543-26/2019 (H11N1)<br>A/ostrich/ZA/543-17/2019 (H11N1)<br>A/ostrich/ZA/0107-21/2019 (H11N1)<br>A/ostrich/ZA/0107-23/2019 (H11N1)	Apr 2018	Nov 2017- Aug 2018	0.79
**PB2**	A/mallard/ZA/0156-38/2019 (H11N9)<br>X<br>A/wild duck/ZL117/2022 (H9N2)<br>A/wild ducks/ZA/ ZL118/2022 (H9N2)<br>A/wild ducks/ZA/ ZL121/2022 (H9N2)<br>A/wild ducks/ZA/ ZL213/2022 (H9N2)<br>A/wild ducks/ZA/ ZL309/2022 (H9N2)<br>A/wild duck/ZL117/2022 (H9N2)<br>A/wild ducks/ZA/ ZL118/2022 (H9N2)<br>A/wild ducks/ZA/ ZL121/2022 (H9N2)<br>A/wild ducks/ZA/ ZL213/2022 (H9N2)<br>A/wild ducks/ZA/ ZL309/2022 (H9N2)<br>A/ostrich/ZA/090581/2020 (H5N2)<br>A/ostrich/ZA/18090431/2018 (H11N1)<br>A/ostrich/ZA/543-31/2019 (H11N1)<br>A/ostrich/ZA/543-20/2019 (H11N1)<br>A/ostrich/ZA/543-26/2019 (H11N1)<br>A/ostrich/ZA/543-17/2019 (H11N1)<br>A/ostrich/ZA/0107-21/2019 (H11N1)<br>A/ostrich/ZA/0107-23/2019 (H11N1)	Jan 2017	Feb 2016-Feb 2018	1
**PB2**	A/pintail/Egypt/MB-D-384C/2015 (H3N6)<br>X<br>All ZA viruses above (PB2 gene)	May 2015	Nov 2014-Sep 2015	0.4
**PB1**	A/wild duck/ZL117/2022 (H9N2)<br>A/wild ducks/ZA/ ZL118/2022 (H9N2)<br>A/wild ducks/ZA/ ZL121/2022 (H9N2)<br>A/wild ducks/ZA/ ZL213/2022 (H9N2)	Nov 2011	Apr 2005-May 2016	0.35

	A/wild ducks/ZA/ ZL309/2022 (H9N2)<br>X<br>A/Common Teal/Buryatia/89i/2019 (H11N1)<br>A/Duck/Mongolia/2019-496/2019 (H11N1)			
**PA**	A/wild duck/ZL117/2022 (H9N2)<br>A/wild ducks/ZA/ ZL118/2022 (H9N2)<br>A/wild ducks/ZA/ ZL121/2022 (H9N2)<br>A/wild ducks/ZA/ ZL213/2022 (H9N2)<br>A/wild ducks/ZA/ ZL309/2022 (H9N2)<br>X<br>A/ostrich/ZA/070595/2020 (H7N1)<br>A/ostrich/ZA/080067/2020 (H7N1)<br>A/ostrich/ZA/653561/2020 (H7N1)	Apr 2019	Jun 2018-Feb 2020	1
**PA**	A/wild duck/ZL117/2022 (H9N2)<br>A/wild ducks/ZA/ ZL118/2022 (H9N2)<br>A/wild ducks/ZA/ ZL121/2022 (H9N2)<br>A/wild ducks/ZA/ ZL213/2022 (H9N2)<br>A/wild ducks/ZA/ ZL309/2022 (H9N2)<br>A/ostrich/ZA/070595/2020 (H7N1)<br>A/ostrich/ZA/080067/2020 (H7N1)<br>A/ostrich/ZA/653561/2020 (H7N1)<br>X<br>A/mallard/Novosibirsk region/988k/2018 (H4N6)	Apr 2017	Mar 2016-May 2018	1
**HA**	A/wild duck/ZL117/2022 (H9N2)<br>A/wild ducks/ZA/ ZL118/2022 (H9N2)<br>A/wild ducks/ZA/ ZL121/2022 (H9N2)<br>A/wild ducks/ZA/ ZL213/2022 (H9N2)<br>A/wild ducks/ZA/ ZL309/2022 (H9N2)<br>X<br>A/Egyptian goose/ZA/TP2118/2021 (H9N2)	Feb 2019	Dec 2015- Dec 2020	1
**HA**	A/wild duck/ZL117/2022 (H9N2)<br>A/wild ducks/ZA/ ZL118/2022 (H9N2)<br>A/wild ducks/ZA/ ZL121/2022 (H9N2)<br>A/wild ducks/ZA/ ZL213/2022 (H9N2)<br>A/wild ducks/ZA/ ZL309/2022 (H9N2)<br>A/Egyptian goose/ZA/TP2118/2021 (H9N2)<br>X<br>A/pheasant/Italy/21VIR2284-1/2021 (H9N2)	Jan 2013	Nov 2005-Feb 2020	1
**NP**	A/wild duck/ZL117/2022 (H9N2)<br>A/wild ducks/ZA/ ZL118/2022 (H9N2)<br>A/wild ducks/ZA/ ZL121/2022 (H9N2)<br>A/wild ducks/ZA/ ZL213/2022 (H9N2)<br>A/wild ducks/ZA/ ZL309/2022 (H9N2)<br>A/Egyptian goose/ZA/TP2118/2021 (H9N2)<br>X<br>A/Egyptian goose/ZA/TP2118/2021 (H9N2)	Oct 2018	Apr 2017-Dec 2019	1
**NP**	A/ostrich/ZA/18090431/2018 (H11N1)<br>X<br>A/ostrich/ZA/543-31/2019 (H11N1)<br>A/ostrich/ZA/543-20/2019 (H11N1)<br>A/ostrich/ZA/543-26/2019 (H11N1)<br>A/ostrich/ZA/543-17/2019 (H11N1)<br>A/ostrich/ZA/0107-21/2019 (H11N1)<br>A/ostrich/ZA/0107-23/2019 (H11N1)	Jul 2018	Mar 2018-Aug 2018	1
**NP**	A/wild duck/ZL117/2022 (H9N2)<br>A/wild ducks/ZA/ ZL118/2022 (H9N2)<br>A/wild ducks/ZA/ ZL121/2022 (H9N2)<br>A/wild ducks/ZA/ ZL213/2022 (H9N2)<br>A/wild ducks/ZA/ ZL309/2022 (H9N2)<br>A/Egyptian goose/ZA/TP2118/2021 (H9N2)<br>X	Jun 2015	Feb 2014-Dec 2016	1

	A/ostrich/ZA/18090431/2018 (H11N1)<br>A/ostrich/ZA/543-31/2019 (H11N1)<br>A/ostrich/ZA/543-20/2019 (H11N1)<br>A/ostrich/ZA/543-26/2019 (H11N1)<br>A/ostrich/ZA/543-17/2019 (H11N1)<br>A/ostrich/ZA/0107-21/2019 (H11N1)<br>A/ostrich/ZA/0107-23/2019 (H11N1)			
**NP**	A/wild duck/ZL117/2022 (H9N2)<br>A/wild ducks/ZA/ ZL118/2022 (H9N2)<br>A/wild ducks/ZA/ ZL121/2022 (H9N2)<br>A/wild ducks/ZA/ ZL213/2022 (H9N2)<br>A/wild ducks/ZA/ ZL309/2022 (H9N2)<br>A/Egyptian goose/ZA/TP2118/2021 (H9N2)<br>A/ostrich/ZA/18090431/2018 (H11N1)<br>A/ostrich/ZA/543-31/2019 (H11N1)<br>A/ostrich/ZA/543-20/2019 (H11N1)<br>A/ostrich/ZA/543-26/2019 (H11N1)<br>A/ostrich/ZA/543-17/2019 (H11N1)<br>A/ostrich/ZA/0107-21/2019 (H11N1)<br>A/ostrich/ZA/0107-23/2019 (H11N1)<br>X<br>A/Anas platyrhynchos/Belgium/17330cls42/2013 (H5N2)	Feb 2012	Mar 2011-Dec 2013	0.12
**NA**	A/wild duck/ZL117/2022 (H9N2)<br>A/wild ducks/ZA/ ZL118/2022 (H9N2)<br>A/wild ducks/ZA/ ZL121/2022 (H9N2)<br>A/wild ducks/ZA/ ZL213/2022 (H9N2)<br>A/wild ducks/ZA/ ZL309/2022 (H9N2)<br>A/Egyptian goose/ZA/TP2118/2021 (H9N2)<br>X<br>A/Egyptian goose/ZA/TP2118/2021 (H9N2)	Jun 2018	Feb 2017-Dec 2019	1
**NA**	/wild duck/ZL117/2022 (H9N2)<br>A/wild ducks/ZA/ ZL118/2022 (H9N2)<br>A/wild ducks/ZA/ ZL121/2022 (H9N2)<br>A/wild ducks/ZA/ ZL213/2022 (H9N2)<br>A/wild ducks/ZA/ ZL309/2022 (H9N2)<br>A/Egyptian goose/ZA/TP2118/2021 (H9N2)<br>X<br>A/wild bird/Uganda/MUWRP-731/2017 (H6N2)	May 2016	Jan 2015-Aug 2017	1
**M**	A/wild duck/ZL117/2022 (H9N2)<br>A/wild ducks/ZA/ ZL118/2022 (H9N2)<br>A/wild ducks/ZA/ ZL121/2022 (H9N2)<br>A/wild ducks/ZA/ ZL213/2022 (H9N2)<br>A/wild ducks/ZA/ ZL309/2022 (H9N2)<br>A/Egyptian goose/ZA/TP2118/2021 (H9N2)<br>X<br>A/Egyptian goose/ZA/TP2118/2021 (H9N2)	Sep 2020	Feb 2020-Feb 2021	1
**M**	A/ostrich/ZA/18090431/2018 (H11N1)<br>X<br>A/ostrich/ZA/543-31/2019 (H11N1)<br>A/ostrich/ZA/543-20/2019 (H11N1)<br>A/ostrich/ZA/543-26/2019 (H11N1)<br>A/ostrich/ZA/543-17/2019 (H11N1)<br>A/ostrich/ZA/0107-21/2019 (H11N1)<br>A/ostrich/ZA/0107-23/2019 (H11N1)	May 2018	Dec 2017-Aug 2018	0.9
**M**	A/wild duck/ZL117/2022 (H9N2)<br>A/wild ducks/ZA/ ZL118/2022 (H9N2)<br>A/wild ducks/ZA/ ZL121/2022 (H9N2)<br>A/wild ducks/ZA/ ZL213/2022 (H9N2)<br>A/wild ducks/ZA/ ZL309/2022 (H9N2)<br>A/Egyptian goose/ZA/TP2118/2021 (H9N2)<br>X	Feb 2018	May 2017-Aug 2018	1

	A/ostrich/ZA/18090431/2018 (H11N1)<br>A/ostrich/ZA/543-31/2019 (H11N1)<br>A/ostrich/ZA/543-20/2019 (H11N1)<br>A/ostrich/ZA/543-26/2019 (H11N1)<br>A/ostrich/ZA/543-17/2019 (H11N1)<br>A/ostrich/ZA/0107-21/2019 (H11N1)<br>A/ostrich/ZA/0107-23/2019 (H11N1)			
**M**	All ZA viruses above (M gene)<br>X<br>A/mallard/Dagestan/1051/2018 (H7N3)	May 2017	Jul 2016-Feb 2018	1
**NS**	A/wild duck/ZL117/2022 (H9N2)<br>A/wild ducks/ZA/ ZL118/2022 (H9N2)<br>A/wild ducks/ZA/ ZL121/2022 (H9N2)<br>A/wild ducks/ZA/ ZL213/2022 (H9N2)<br>A/wild ducks/ZA/ ZL309/2022 (H9N2)<br>A/Egyptian goose/ZA/TP2118/2021 (H9N2)<br>X<br>A/Egyptian goose/ZA/TP2118/2021 (H9N2)	Sep 2020	Nov 2019-Feb 2021	1
**NS**	A/wild duck/ZL117/2022 (H9N2)<br>A/wild ducks/ZA/ ZL118/2022 (H9N2)<br>A/wild ducks/ZA/ ZL121/2022 (H9N2)<br>A/wild ducks/ZA/ ZL213/2022 (H9N2)<br>A/wild ducks/ZA/ ZL309/2022 (H9N2)<br>A/Egyptian goose/ZA/TP2118/2021 (H9N2)<br>X<br>A/ostrich/ZA/070595/2020 (H7N1)<br>A/ostrich/ZA/080067/2020 (H7N1)<br>A/ostrich/ZA/653561/2020 (H7N1)	Sep 2018	Dec 2016-Sep 2019	1
**NS**	A/wild duck/ZL117/2022 (H9N2)<br>A/wild ducks/ZA/ ZL118/2022 (H9N2)<br>A/wild ducks/ZA/ ZL121/2022 (H9N2)<br>A/wild ducks/ZA/ ZL213/2022 (H9N2)<br>A/wild ducks/ZA/ ZL309/2022 (H9N2)<br>A/Egyptian goose/ZA/TP2118/2021 (H9N2)<br>A/ostrich/ZA/070595/2020 (H7N1)<br>A/ostrich/ZA/080067/2020 (H7N1)<br>A/ostrich/ZA/653561/2020 (H7N1)<br>X<br>A/mallard/Ukraine/AN-221-13-01/2020 (H7N2)	Aug 2016	Dec 2014-Jun 2018	0.99
